# Synchronous eyelid oncocytoma and conjunctival intraepithelial neoplasia

**DOI:** 10.4322/acr.2020.235

**Published:** 2021-01-28

**Authors:** Zachariah Chowdhury, Benjamin Nongrum, Chandana Rajbongshi

**Affiliations:** 1 North Eastern Indira Gandhi Regional Institute of Health & Medical Sciences, Department of Pathology, Shillong, Meghalaya, India; 2 North Eastern Indira Gandhi Regional Institute of Health & Medical Sciences, Department of Ophthalmology, Shillong, Meghalaya, India

**Keywords:** Eyelid Neoplasms, Papilloma, Pterygium

## Abstract

Oncocytoma of the eyelid is a rare neoplasm. Oncocytoma associated with an ocular surface squamous neoplasm, namely conjunctival intraepithelial neoplasia, is very hard to find in the literature. Herein we report a case of a 53-year-old male who presented with a swelling in the right lower lid over the last 6 years, along with a growth in the conjunctiva of the same eye for the last 2 years and encroaching upon the cornea for the last 4 months. Excision biopsy of the lower lid mass showed histopathological features consistent with oncocytoma. The conjunctival tissue revealed conjunctival intraepithelial neoplasia 3 (severe dysplasia). This case documents a rare synchronous dual ocular neoplasia, a very unlikely coexistence of oncocytoma with conjunctival intraepithelial neoplasia.

## INTRODUCTION

Oncocytes are defined as modified epithelial cells with characteristic fine, granular, and eosinophilic cytoplasm containing an excessive number of mitochondria. Tumors composed of oncocytes, known as oncocytomas, have been described in various organs, including the kidneys, adrenal glands, thyroid, parathyroid, pancreas, and respiratory tract, among others.^1^ ^-^ ^3^ In 1941, Radnot^4^ first reported an oncocytic tumor of the ocular adnexa, and since then, it is rarely observed. The most common ocular site is the caruncle, followed by the lacrimal gland, conjunctiva, eyelid margin, and lacrimal sac. The estimated incidence of histopathologically-proven oncocytic lesions in the ocular region is 0.3 per million per year.^2^ ^,^ ^5^ The association of oncocytoma of the eyelid with ocular surface squamous neoplasia, and conjunctival intraepithelial neoplasia has not been documented to date.

## CASE REPORT

**Figure 1:**
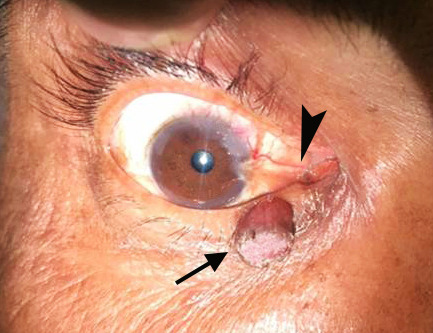
Gross view of the swelling in the right lower eyelid (arrow) along with a fleshy growth in the conjunctiva (arrowhead) of the same eye encroaching upon the cornea.

A 53-year-old male presented with a swelling in the right lower lid gradually increasing in size over the last 6 years, along with a growth in the conjunctiva of the same eye during the last 2 years, accompanied by difficulty in vision since last 4 months (Figure 1).

**Figure 2:**
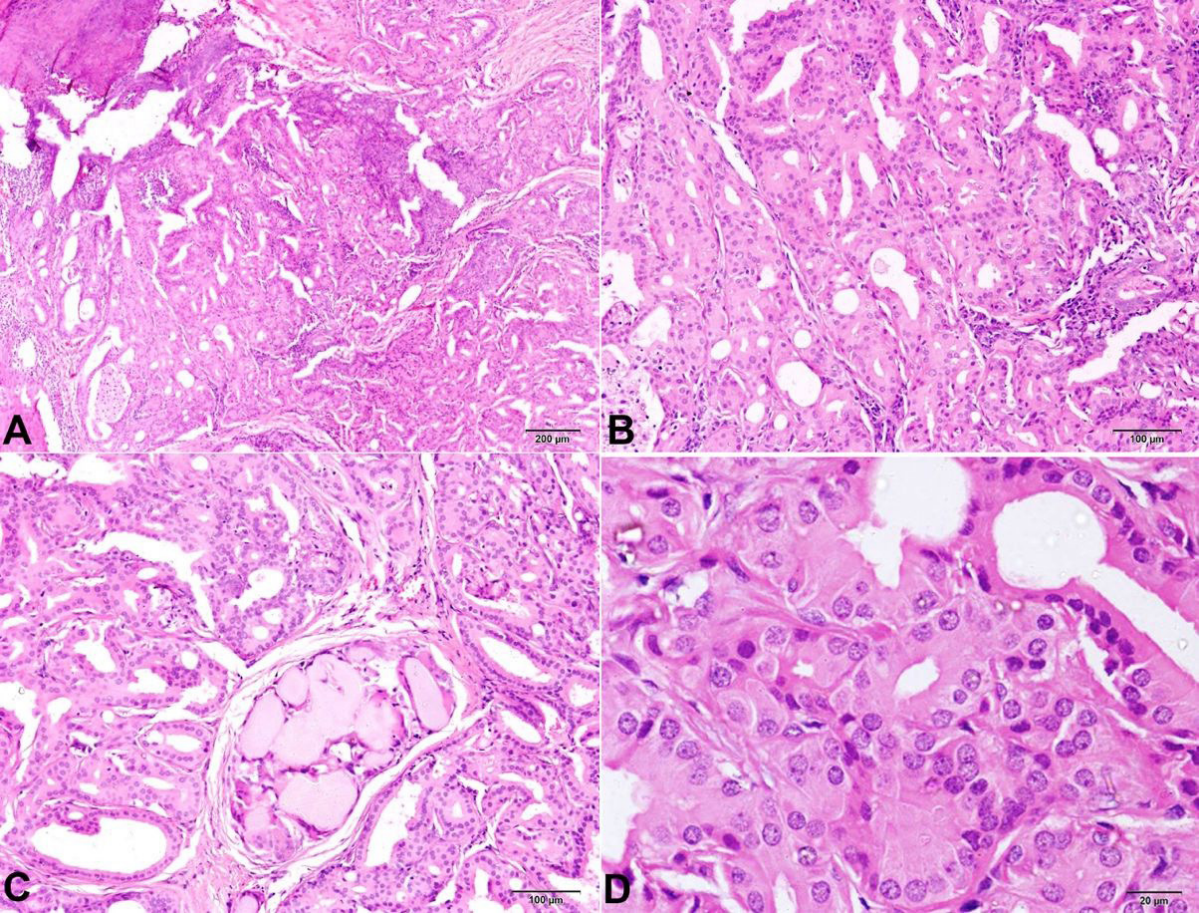
Photomicrograph of the eyelid lesion demonstrates glandular spaces lined by tall cells exhibiting uniform round to oval nuclei, fine chromatin, and abundant granular eosinophilic cytoplasm (oncocytes). At places, there was evidence of secretion within the glandular spaces and interspersed lymphoplasmacytic inflammation (H&E, **A –** 4X; **B –** 10X; **C –** 10X; **D –** 40X).

**Figure 3:**
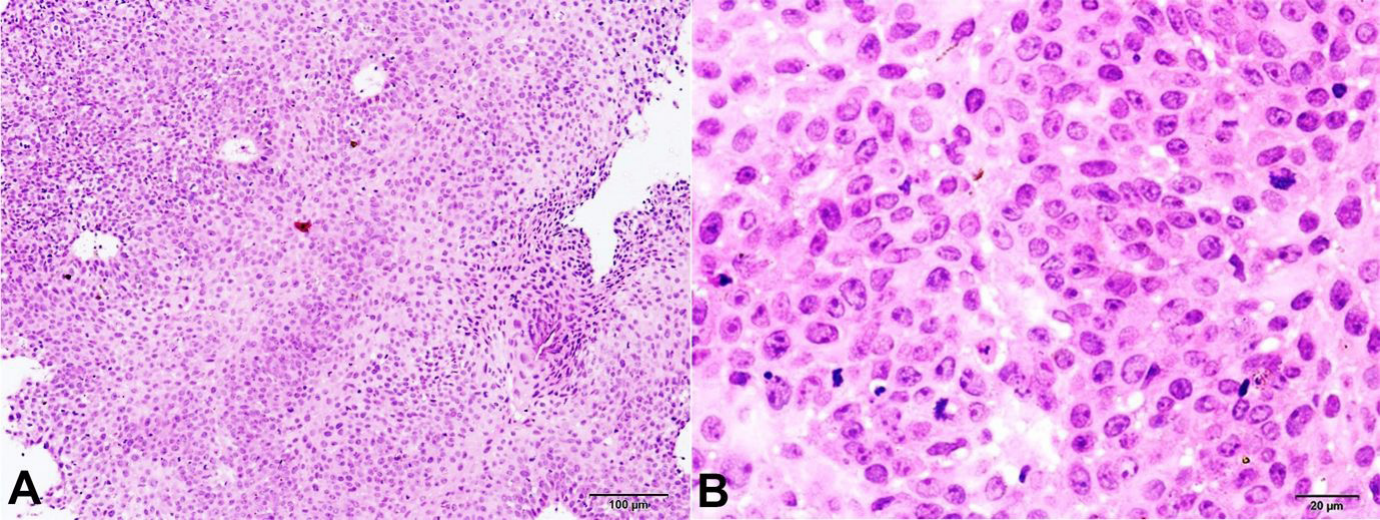
Histopathology of the conjunctival lesion exhibits a superficial biopsy comprising only acanthotic stratified squamous epithelium displaying severe dysplasia (H&E, **A –** 10X; **B –** 40X).

He did not have any other subjective symptoms such as pain, discharge, lacrimation, bleeding, or change in color. There was no history of trauma, or eye surgery. On examination, a round, well-circumscribed, mobile, elevated lesion measuring 2.5 × 1.5 cm was seen in the lower lid of the right eye. Differential diagnosis of papilloma was clinically considered. Another grey white growth measuring 0.15 cm in diameter was noted in the conjunctiva, which overhung the cornea. Both the lesions were excised “en masse” and sent for histopathological examination (HPE). HPE of the eyelid lesion showed a piece of tissue lined by non-ulcerated hyperplastic, hyperkeratotic stratified squamous epithelium with the underlying tissue displaying a well-demarcated mass composed of glandular spaces separated by thin vasculature. The glandular spaces were lined by tall cells exhibiting uniform round to oval nuclei, fine chromatin, and abundant granular eosinophilic cytoplasm (oncocytes). At places, there was evidence of secretion within the glandular spaces and interspersed lymphoplasmacytic inflammation. Mitotic figures were not seen. The microscopic features were consistent with a diagnosis of oncocytoma (Figure 2). The HPE of the conjunctival lesion showed a superficial biopsy comprising only the acanthotic stratified squamous epithelium, which exhibited severe dysplasia. The basement membrane was not visualized in its entirety, and there was no subepithelial tissue to comment on an invasion. A diagnosis of conjunctival intraepithelial neoplasia 3 (severe dysplasia) was proffered. Adjuvant topical chemotherapy with 5-fluorouracil was prescribed to the patient to minimize recurrence (Figure 3).

## DISCUSSION

Oncocytomas are composed of epithelial cells with a distinctive appearance characterized by a large size and abundant, granular, eosinophilic cytoplasm, known as oncocytes. Electron microscopy of oncocytomas revealed a cytoplasm packed with abnormal and possibly dysfunctional mitochondria of varying sizes and shapes with irregular and fragmented cristae.^6^ More than half of the reported cases of the ocular oncocytoma is the caruncle. Oncocytoma of the eyelid margin is rare, with only a handful of cases previously reported. The average age is 68 years, and 60% were female. The duration of symptoms ranged from two months to five years, with no evidence of recurrence reported after excision.^7^ ^,^ ^8^

Clinically, oncocytomas have been described as red-brown or yellow-tan lesions that are elevated and, at times, lobulated, or cystic.^8^ Close differential diagnoses of such tumors in the eyelid include squamous papilloma, capillary hemangioma, melanocytic nevi, pyogenic granuloma, basal cell carcinoma among others. Histopathologically, oncocytomas are characterized by a proliferation of large, uniform, polygonal epithelial cells that exhibit abundant, fine granular eosinophilic cytoplasm and contain dark round to ovoid paracentral nuclei (oncocytes).^2^ The oncocytes form solid cords and tubular structures that occasionally display papillary infoldings as well as the formation of large cystic spaces.^8^

The origin of oncocytomas in the ocular adnexa remains a matter of debate. Rodgers et al.,^9^ and Fukuo et al.^10^ suggested that oncocytomas of the eyelid arose from an adjacent apocrine gland of the eyelid margin (gland of Moll). However, in a more recent study, Østergaard et al.^2^ examined the cytokeratin (CK) profile of oncocytic lesions of the ophthalmic region to investigate the origin of these tumors. He concluded that oncocytic lesions of the ophthalmic region demonstrate a CK profile similar to lacrimal and accessory lacrimal gland duct elements; thus, supporting the theory that oncocytic lesions are ductal neoplasms originating from lacrimal and accessory lacrimal glands.

The association of oncocytomas of the eyelid with conjunctival intraepithelial neoplasia (CIN) is quite rare, and the authors did not find any other case in the English literature. CIN is one entity under the umbrella of ocular surface squamous neoplasia (OSSN), which encompasses a varied spectrum of entities involving abnormal growth of dysplastic squamous epithelial cells on the surface of the eye, including the invasive squamous cell carcinoma (SCC) of the conjunctiva and cornea.^11^ This lesion has a multifactorial etiology with the interplay of several factors like exposure to ultraviolet radiation, various chemical carcinogens, and viral infections. The epithelium of the ocular surface is exposed to the environment. Thus, it is quite susceptible to infections, particularly when protective barriers of mucin, tears, and superficial cellular layer are compromised. Human papillomavirus has mainly been implicated in the pathogenesis of ocular surface diseases, such as conjunctival papilloma, and carcinoma of the lacrimal sac, conjunctival pterygium, OSSN including CIN, and even squamous cell carcinoma of the conjunctiva.^12^ According to epidemiologic studies, the prevalence of OSSN is estimated to range from <0.2 cases/million/year (UK, 1996) to 35 cases/million/year (Uganda, 1992).^13^ ^,^ ^14^

Clinically OSSN has myriad presentations. It usually appears as a sessile, fleshy, elevated lesion adjacent to the limbus in the inter-palpebral region. The presentation of CIN and invasive SCC is very similar; thus, making the clinical differentiation difficult.^11^ The gold standard for the diagnosis of OSSN is the histopathological evaluation of the lesion after an incisional or excisional biopsy. Histologically, the epithelium exhibits hyperplasia, loss of the goblet cells, loss of the normal cell polarity, nuclear hyperchromasia and pleomorphism, and mitotic figures. There is often surface keratinization, correlating with the leukoplakia observed clinically. Dyskeratosis may also be seen, along with a chronic inflammatory response in the substantia propria. The most important assessment to be made histologically in OSSN is whether the neoplasia is contained by the basement membrane (i.e., intraepithelial or in situ), or whether neoplastic cells have traversed the epithelial basement membrane and invaded the stroma. For lesions contained by the basement membrane, the term conjunctival intraepithelial neoplasia (CIN) may be used. CIN refers to varying degrees of conjunctival epithelial dysplasia. For instance, CIN I represents a mild disease, CIN II refers to a moderate disease, and CIN III suggests near full-thickness (more than 2/3rds of the epithelial thickness) epithelial dysplasia; however, the surface maturation is preserved. CIN that involves the entire epithelium, is referred to as carcinoma-in-situ.^15^ CIN can progress to invasive squamous cell carcinoma with the destruction of the epithelial basement membrane and extension into the underlying stromal tissue.^11^

Reactive, regenerative, or reparative squamous epithelium (for example, in response to trauma, inflammation, or infection) may manifest atypical cytology. Such changes should be studied with caution, and distinguished from CIN. Other ocular surface lesions that can clinically mimic OSSN, such as actinic keratosis, pterygium, pinguecula, and actinic granuloma, can be easily distinguished from OSSN by studying the histomorphology. OSSN can uncommonly coexist with pterygium. Therefore, a careful study of pterygium specimens should be executed to exclude dysplastic changes.^16^ Although regional lymph node metastasis is not as common as it is with squamous carcinomas of the skin or other sites, dissemination and death can occur.

Complete surgical resection of the eyelid oncocytoma with primary repair of the resulting defect was performed in our case, as is the treatment of choice. As benign oncocytomas may recur or transform into malignant oncocytomas,^17^ the patient was put on long-term follow-up for local recurrence. As for OSSN, management modalities range from complete excision in well-delineated tumors to chemotherapy in diffuse unresectable lesions.^18^ ^-^ ^20^ Surgical management is aimed at removing the entire tumor in one piece and is indicated for tumors that occupy ≤ 4 clock hours of the limbus and have a basal diameter of less than 15mm. The surgical approach follows the traditional “no-touch” technique, which minimizes seeding of the tumor during surgery, and recommends 3-4 mm macroscopically clear margins. The main risks associated with surgery are limbal stem cell deficiency, scarring, pyogenic granulomas, infection, and damage to the sclera or retina from excessive cryotherapy. The limitation of surgery is that it only removes the macroscopically visible tumor. If the surgical margins are found to be involved on histology, adjuvant topical chemotherapy can be given to minimize recurrence.^21^ ^-^ ^23^ Medical alternatives in the form of topical applications like 5-Fluorouracil (5FU) and mitomycin C (MMC) have been extensively reported in the literature.^11^ In 1994, Maskin was the first to report the use of topical interferon (IFNa2b) in a multi-focal limbal OSSN.^24^ Over the past decade, several authors have reported the beneficial effects of IFNa2b in the treatment of OSSN.^25^ The overall prognosis of both these neoplasms is good.

To conclude, eyelid oncocytomas though rare, should be included in the differential diagnosis of eyelid tumors. The present case is an unusual example of the concurrent existence of dual ocular pathology. It underlines the significance of histopathology as the gold standard in the diagnoses of various grades and types of OSSN. A high degree of clinical suspicion with appropriate tests facilitates early diagnosis and curative surgical excision.
